# Systemic Lupus Erythematosus and Antineutrophil Cytoplasmic Antibody-Associated Vasculitis Overlap Syndrome in Patients With Biopsy-Proven Glomerulonephritis

**DOI:** 10.1097/MD.0000000000003748

**Published:** 2016-06-03

**Authors:** Pierre-Andre Jarrot, Laurent Chiche, Baptiste Hervier, Laurent Daniel, Vincent Vuiblet, Nathalie Bardin, Daniel Bertin, Benjamin Terrier, Zahir Amoura, Emmanuel Andrés, Eric Rondeau, Mohamed Hamidou, Jean-Loup Pennaforte, Philippe Halfon, Eric Daugas, Bertrand Dussol, Xavier Puéchal, Gilles Kaplanski, Noemie Jourde-Chiche

**Affiliations:** From the Department of Internal Medicine and Clinical Immunology (PAJ, GK), AP-HM Hôpital de La Conception; Inserm UMR-S 1076 Vascular Research Center of Marseille (PAJ, GK, NJ-C), Aix-Marseille Université; Department of Internal Medicine (LC, PH), Hôpital Européen de Marseille, Marseille; Reference Center for Systemic Lupus Erythematosus (BH, ZA), Department of Internal Medicine, AP-HP Hôpital Pitie-Salpêtrière, Paris; Department of Pathology (LD), AP-HM Hôpital de La Timone, Aix-Marseille Université; Department of Pathology (VV), Paul Bouin Laboratory, Reims, France; Laboratory of Immunology (NB, DB), AP-HM Hôpital de la Conception, Aix-Marseille Université, Marseille; French Vasculitis Study Group (BT, XP), Department of Internal Medicine, National Reference Center for Necrotizing Vasculitis, AP-HP Hôpital Cochin, University Paris-Descartes; Department of Internal Medicine (EA), Hôpital Civil, Strasbourg; Department of Nephrology (ER), AP-HP Hôpital Tenon, Paris; Department of Internal Medicine (MH), Hôpital Hôtel-Dieu, Nantes; Department of Internal Medicine (J-LP), Hôpital Robert Debré, Reims Université, Reims; Groupe Coopératif sur le Lupus Rénal (ED), Department of Nephrology, AP-HP Hôpital Bichat, Paris; and Department of Nephrology (BD, NJ-C), AP-HM Hôpital Conception, Aix-Marseille Université, Marseille, France.

## Abstract

The aim of the study was to report the clinical, biological, and pathological characteristics of patients with glomerulonephritis (GN) secondary to systemic lupus erythematosus (SLE)/antineutrophil cytoplasmic antibodies (ANCA)-associated vasculitis (AAV) overlap syndrome.

A nationwide survey was conducted to identify cases of SLE/AAV overlap syndrome. Data were collected from SLE and AAV French research groups. Inclusion criteria were diagnosis of both SLE and AAV according to international classification criteria and biopsy-proven GN between 1995 and 2014. Additional cases were identified through a systematic literature review. A cohort of consecutive biopsy-proven GN was used to study the prevalence of overlapping antibodies and/or overlap syndrome.

The national survey identified 8 cases of SLE/AAV overlap syndrome. All patients were female; median age was 40 years. AAV occurred before SLE (n = 3), after (n = 3), or concomitantly (n = 2). Six patients had rapidly progressive GN and 3/8 had alveolar hemorrhage. All patients had antinuclear antibodies (ANA); 7/8 had p-ANCA antimyeloperoxidase (MPO) antibodies. Renal biopsies showed lupus nephritis (LN) or pauci-immune GN. Remission was obtained in 4/8 patients. A literature review identified 31 additional cases with a similarly severe presentation. In the GN cohort, ANCA positivity was found in 30% of LN, ANA positivity in 52% of pauci-immune GN, with no correlation with pathological findings. The estimated prevalence for SLE/AAV overlap syndrome was 2/101 (2%).

In patients with GN, SLE/AAV overlap syndrome may occur but with a low prevalence. Most patients have an aggressive renal presentation, with usually both ANA and anti-MPO antibodies. Further studies are needed to assess shared pathogenesis and therapeutic options.

## Abstract 2

Supplemental Digital Content is available in the text

## INTRODUCTION

Systemic lupus erythematosus (SLE) is a chronic and severe autoimmune disease characterized by the presence of a wide range of serum autoantibodies, such as antinuclear (ANA) and antidouble-stranded deoxyribonucleic acid (anti-dsDNA) antibodies.^1^ SLE's clinical presentation is heterogeneous and can display a broad spectrum of manifestations, including lupus nephritis (LN) in 30% to 60% of patients and vasculitis in 11% of patients.^2–4^ LN is characterized by glomerular immune-complex deposits.^5^ In contrast, renal involvement of antineutrophil cytoplasmic antibody (ANCA)-associated vasculitis (AAV) is typically a pauci-immune crescentic glomerulonephritis (GN).^6^ Crescent formation, which is the hallmark of renal vasculitis, is however frequently encountered in severe forms of LN.^7^ In addition, ANCA are found in up to 20% of SLE patients, although their pathogenic role in the formation of crescents or necrotizing vasculitis remains unclear.^8^ Some patients fulfilling both SLE and AAV classification criteria were recently defined as having SLE/AAV overlap syndrome.^9^ However, renal pathological details were often unavailable in these cases.

The aims of this study were (i) to describe SLE/AAV overlap cases with biopsy proven GN, and (ii) to assess the prevalence of overlapping autoantibodies and SLE/AAV overlap syndrome in an independent cohort of patients with biopsy-proven GN.

## METHODS

### Ethical Statement

This retrospective observational study was conducted according to the principles expressed in the Declaration of Helsinki. The nationwide survey was conducted though the registries of French reference centers approved by the “Commission Nationale de l’Informatique et des Libertés” (CNIL, registration number 1884512). The cohort analysis was conducted through a biobank approved by the French government (Cellule de Bioéthique, Ministère de l’Enseignement Supérieur et de la Recherche). All patients included in this biobank (DC-2012-1704) gave their written informed consent prior to the collection of data and of samples.

### Nationwide Survey

A retrospective nationwide survey was conducted to collect cases of SLE/AAV overlap syndrome with biopsy-proven GN, diagnosed between 1995 and 2014, through the databases of the “French Vasculitis Study Group” (FVSG), the “Club Rhumatismes et Inflammation” (CRI), and the “Groupe Coopératif sur le Lupus Rénal” (GCLR). Medical charts were reviewed independently by 2 experts to validate the diagnosis of overlap syndrome. Inclusion criteria were as follows: adult or pediatric patients fulfilling (successively or concomitantly) both the American College of Rheumatology (ACR) 1997 SLE classification criteria^10^ and the revised Chapel Hill 2012 AAV definition criteria,^11^ associated with biopsy-proven LN or pauci-immune GN. Exclusion criteria were having positive antiglomerular basement membrane (GBM) antibodies, post-infectious GN, IgA vasculitis (Henoch–Schonlein purpura), or crescentic IgA nephropathy, mixed cryoglobulinemia associated GN and GN in the context of viral infections.

Clinical manifestations of the initial disease (SLE, AAV, or inaugural overlap syndrome), as well as at the time when overlap syndrome was diagnosed, were recorded: general symptoms (fever, weight loss); cutaneous lesions; ear–nose–throat involvement; ophthalmologic, articular, neurologic, cardiac, pulmonary, vascular involvement. Disease activity at the time when overlap syndrome was diagnosed was assessed by both the SLE Disease Activity Index (SLEDAI)^12^ and the Birmingham Vasculitis Activity Score (BVAS).^13^ Laboratory results were recorded at the time when overlap syndrome was diagnosed: blood leukocyte count, platelet count, hemoglobin, markers of hemolysis, Coombs test, C-reactive protein, serum creatinine, 24-h proteinuria and urinary sediment, ANA, antiextractable nuclear antigens, anti-dsDNA, antiphospholipid antibodies (anticardiolipin, anti-β2GP1, and/or lupus anticoagulant), C3 and C4 complement fractions. Results of the kidney biopsy performed at the time when overlap syndrome was diagnosed included findings from light microscopy and immunofluorescence (IF). LN was classified according to the 2003 revised classification of ISN/RPS.^5^ Pauci-immune GN was classified according to the 2010 classification.^6^ The percentages of sclerotic and crescentic glomeruli and the presence of endocapillary proliferation were detailed for each biopsy. The presence of subendothelial, mesangial, and subepithelial immune complex deposits (IgG, IgM, IgA, C3, or C1q) were semiquantitatively assessed as (–), (±), +, 2+, 3+, 4+. The therapeutic regimen used to treat the initial disease and overlap syndrome was also recorded. Response to treatment was evaluated at M6, M12, M18, and M24 after overlap syndrome was diagnosed according to the remission criteria for the most recent disease, as detailed in the Supplemental Methods Appendix.

### Literature Review

Clinical, biological, and pathological characteristics of the patients identified though the national survey were compared with cases of SLE/AAV overlap syndrome. These were identified through a systematic literature review using the following MESH terms within MEDLINE (National Library of Medicine, Bethesda, MD): systemic vasculitis, AAV, SLE, LN, ANCA, Wegener's granulomatosis, granulomatosis with polyangiitis (GPA), microscopic polyangiitis (MPA), overlap, and association.

### Cohort Analysis and Estimated Prevalence

The prevalence of overlapping antibodies and/or SLE/AAV overlap syndrome was assessed in an independent cohort of consecutive patients with LN or pauci-immune GN diagnosed between 2005 and 2014. Inclusion criteria were being an adult or pediatric patient with biopsy-proven LN or pauci-immune GN and with available immunological data (ANA, anti-dsDNA, ANCA, Indirect IF [IIF], anti-MPO, anti-PR3). Exclusion criteria were similar to those of the nationwide survey. Pathological and immunological analytical methods are detailed in the Supplemental Methods Appendix.

### Statistics

Statistical software GraphPad Prism (GraphPad software, San Diego, CA) was used for the analyses. For the descriptive analyses, results were expressed as frequencies and percentages of categorical variables. Means and standard deviations or medians and ranges were used for continuous variables with asymmetrical distributions. The groups of patients with GN with/without overlapping antibodies were compared using the chi-squared test for frequencies and Student's *t* test for continuous variables. Statistical significance was considered at *P* < 0.05.

## RESULTS

### Nationwide Survey

The nationwide survey allowed identification of 8 patients (all female) with SLE/AAV overlap syndrome and biopsy-proven LN or pauci-immune GN, of whom 3 (patients 2, 6, and 8) had already been reported.^9^ The characteristics of these patients are summarized in Table 1  .

**TABLE 1:**
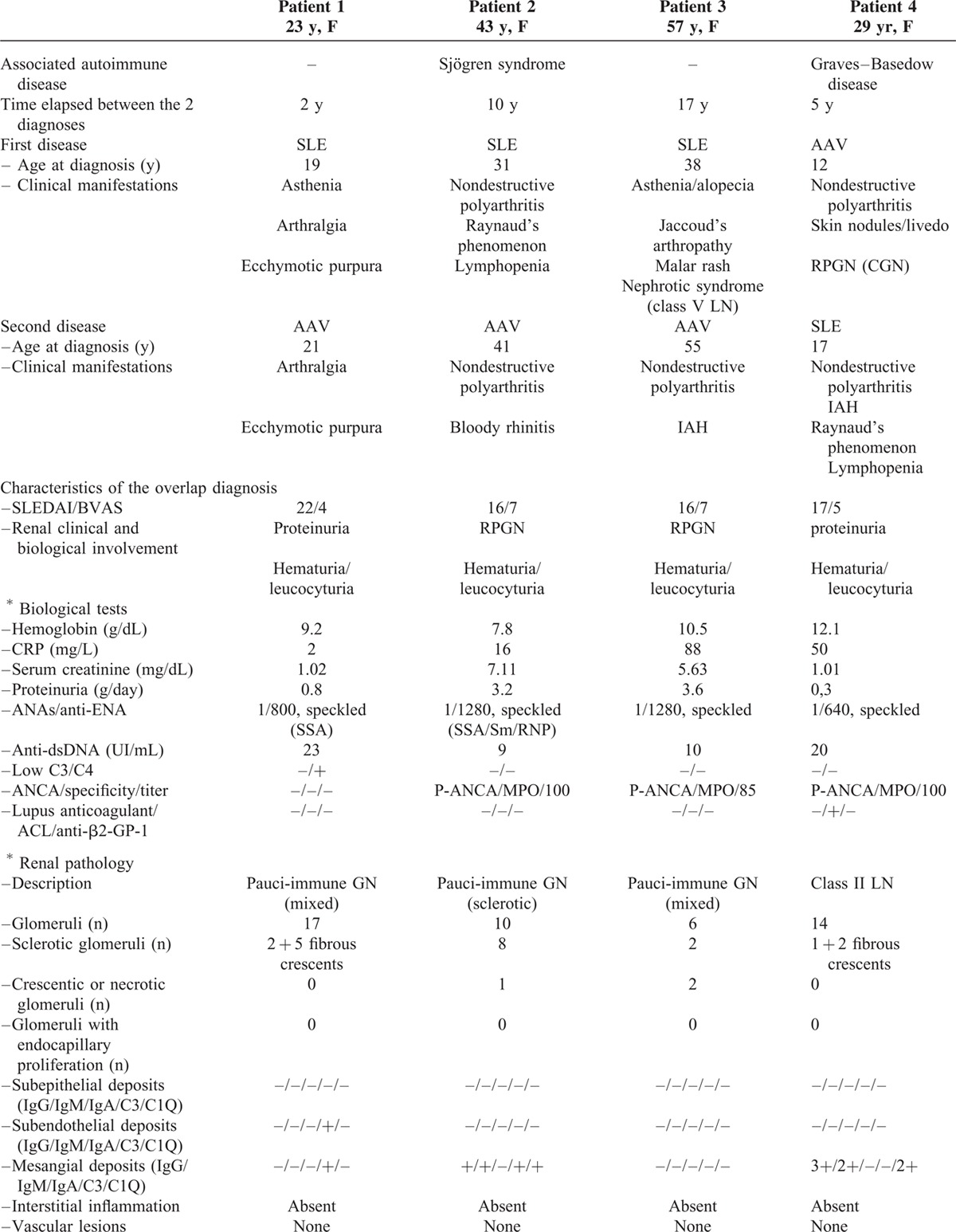
Characteristics of Patients With the SLE/AAV Overlap Syndrome

**TABLE 1 (Continued):**
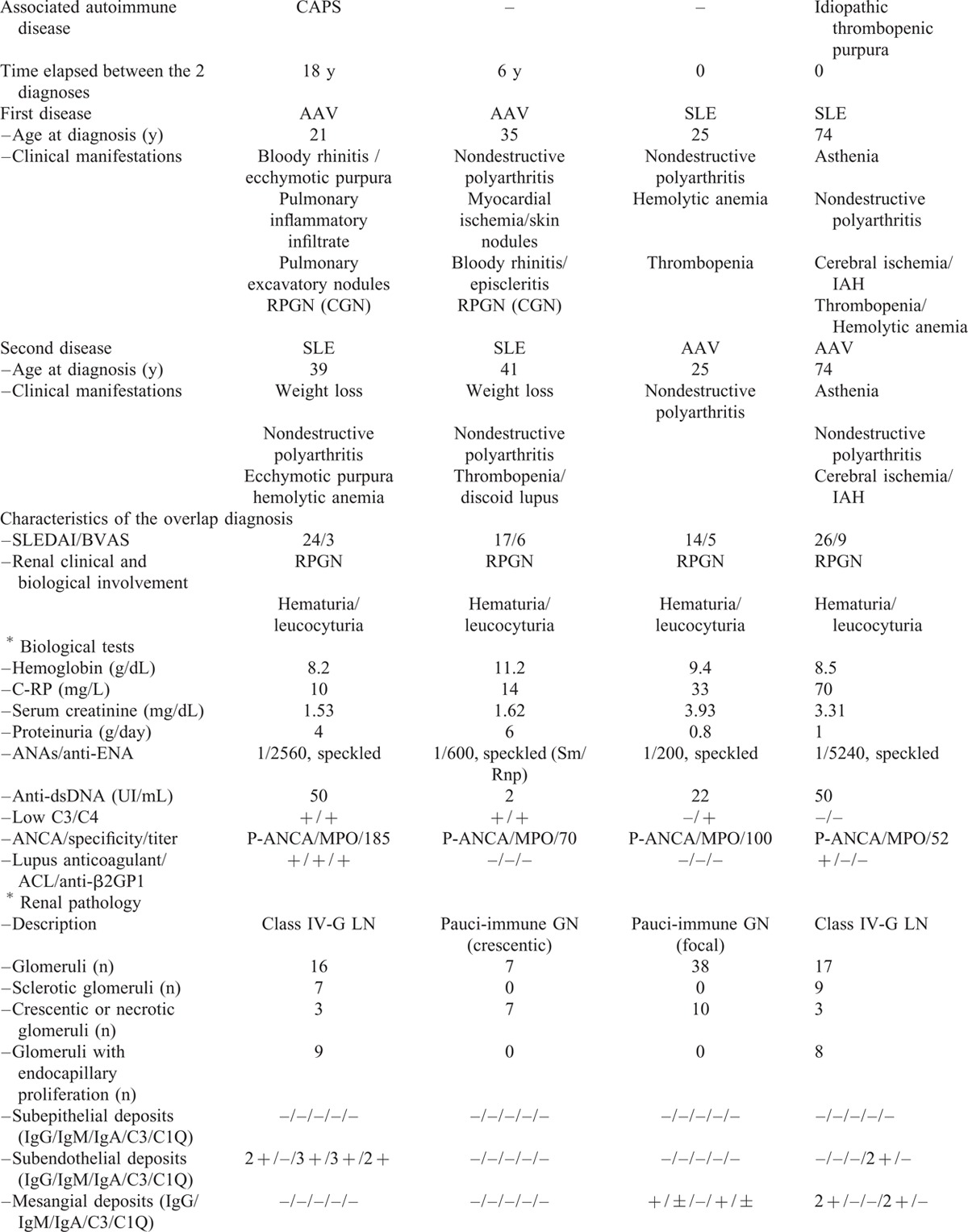
Characteristics of Patients With the SLE/AAV Overlap Syndrome

**TABLE 1 (Continued):**
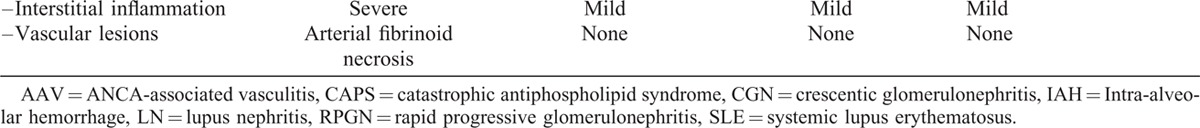
Characteristics of Patients With the SLE/AAV Overlap Syndrome

SLE was the inaugural disease in 3 patients, whereas AAV was first diagnosed in 3 others, and SLE and AAV occurred concomitantly in 2 patients. Median age at the time when overlap syndrome was diagnosed was 40 years (range 17–74).

SLE manifestations were articular (8/8), hematological (6/8), and muco-cutaneous (6/8). AAV manifestations included articular (7/8), muco-cutaneous (4/8), pulmonary (4/8), and ear–nose–throat (3/8) involvement. Myocardial ischemia, cerebral infarction, and episcleritis were all reported in 1 patient. One patient (patient 3) presented with intra-alveolar hemorrhage (IAH) and cerebral infarction. One patient (patient 6) had a past history of nephrotic syndrome due to class V LN, 17 years before the occurrence of rapidly progressive GN with IAH, which led to a diagnosis of MPO-associated AAV with pauci-immune GN. Four patients had a past history of another autoimmune disease that consisted of Sjögren's syndrome, Graves–Basedow disease, idiopathic thrombocytopenic purpura, and/or antiphospholipid syndrome.

At the time when overlap syndrome was diagnosed, median SLEDAI score was 17 (range: 14–26) and median BVAS was 6 (range: 3–9). The most frequent presentation was rapidly progressive GN (6/8 cases), with IAH in 3 patients. Median serum creatinine was 2.47 mg/dL (range: 1.01–7.11). All patients had positive ANA, 5/8 had anti-dsDNA, and 7/8 patients had anti-MPO antibodies.

Pathological diagnosis was pauci-immune GN in 5/8 patients (crescentic; focal; sclerotic, respectively, in 1 case and mixed in 2 cases); it was LN for the other 3 cases (class IV-G in 2 cases, both with crescents, and class II with fibrous crescents in 1 case). The pathological results are provided in Table 1  . Some cases of LN showed prominent crescentic glomeruli, with mild endocapillary proliferation and little immune deposition, whereas some cases of pauci-immune GN showed moderate mesangial immunoglobulin deposition and proliferation.

An induction immunosuppressive regimen for the overlap syndrome included corticosteroids and cyclophosphamide in 7/8 patients, associated with plasma exchanges in 3/8 patients. All patients received a maintenance treatment that included steroids. The detailed therapeutic regimens for each patient are shown in Table 2. Median follow-up after treatment of overlap syndrome was 2 (range: 2–21) years. During the follow-up, 1 patient died from hepatocellular carcinoma at M7, remission was obtained in 4 patients, and 3 patients developed chronic kidney disease (stage 3 for 2 patients, stage 5 requiring hemodialysis and kidney transplantation for 1 patient). A relapse of AAV occurred in2 patients after 4 and 5 years. Serious infectious adverse events occurred in 2 other patients (1 bacterial pneumonia and 1 infectious endocarditis).

**TABLE 2:**
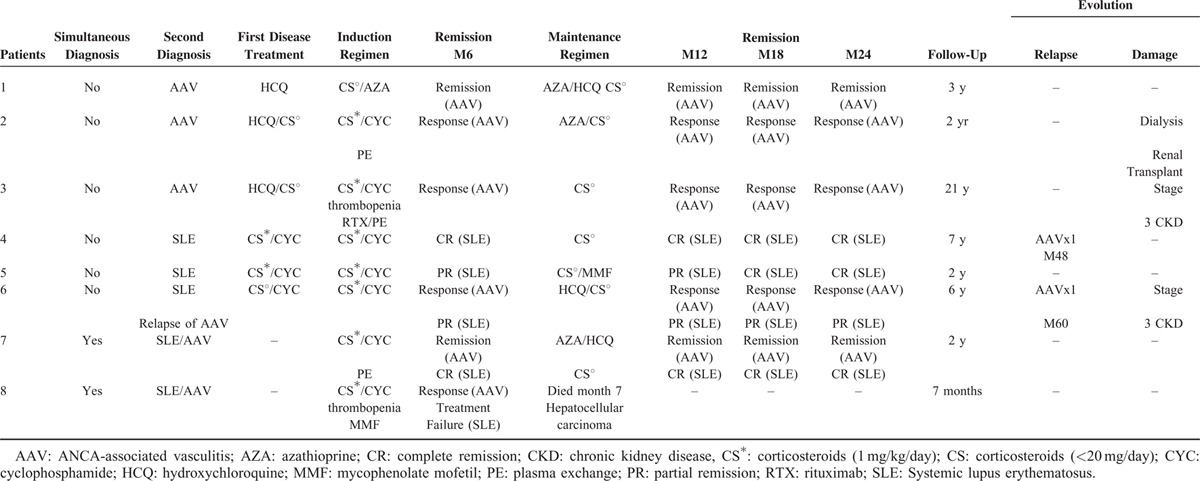
Treatment and Outcome of Patients With the SLE/AAV Overlap Syndrome

### Systematic Literature Review

A systematic literature review allowed the identification of 31 additional cases of SLE/AAV overlap syndrome with renal involvement (excluding the 3 French patients included in the present cohort), among which 30 were documented by a renal biopsy.^7,14–23^ Their characteristics are described in Table 3 and compared with those of the 8 patients from the present study. Consistent with our study, patients were mostly female and presented with rapidly progressive GN (30/31). P-ANCA antibodies were detected in 30/31, anti-MPO antibodies in 21/26 patients, whereas no patient had anti-PR3 antibodies. The mean time interval between the 2 diseases was shorter in the literature than in our patients, with more frequent concomitant diagnoses. Likewise, treatment of overlap syndrome comprised corticosteroids and immunosuppressive drugs.

**TABLE 3:**
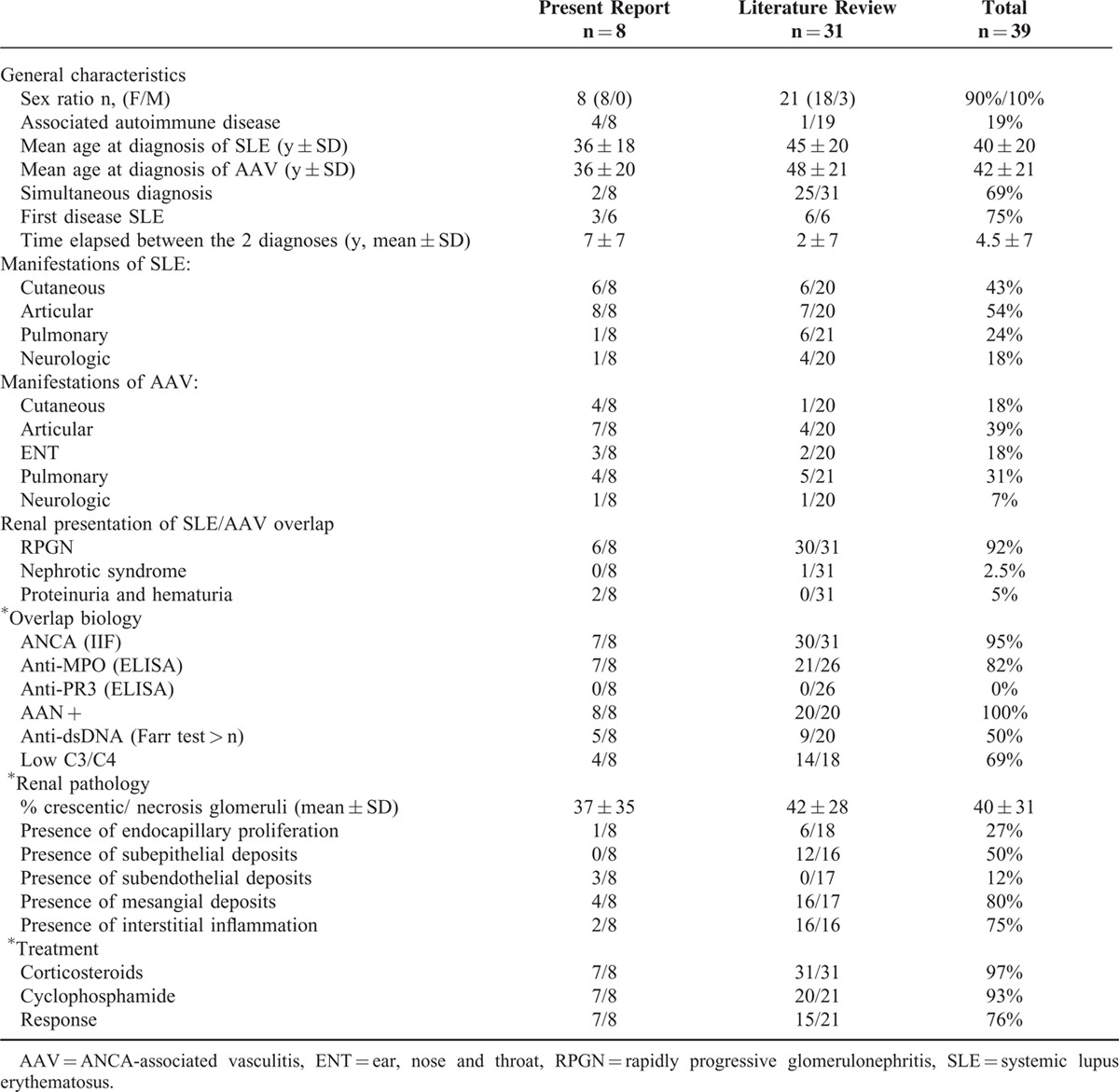
Patients With the SLE/AAV Syndrome From the Present Report and From Previous Reports in the Literature

Overall, in the 39 cases, remission was achieved in 76% (22/29); 4 patients died of infectious adverse events and 1 of cancer (hepatocellular carcinoma). Considering patients for whom a previous treatment was reported, 5/27 (including patient 7) had received drugs potentially associated with drug-induced SLE or vasculitis (hydralazine n = 3, thioridazine n = 1, etanercept n = 1), among which 3 cases had positive antihistone antibodies.

### Prevalence of Overlapping Auto-Antibodies and of SLE/AAV Overlap Syndrome

Among a cohort registry of consecutive kidney biopsies corresponding to LN and pauci-immune GN, complete immunological data were available for 110 biopsies from 101 patients. There were 44 LN (40%) in 40 patients and 66 cases of pauci-immune GN (60%) in 61 patients; the characteristics are described in Table 4.

**TABLE 4:**
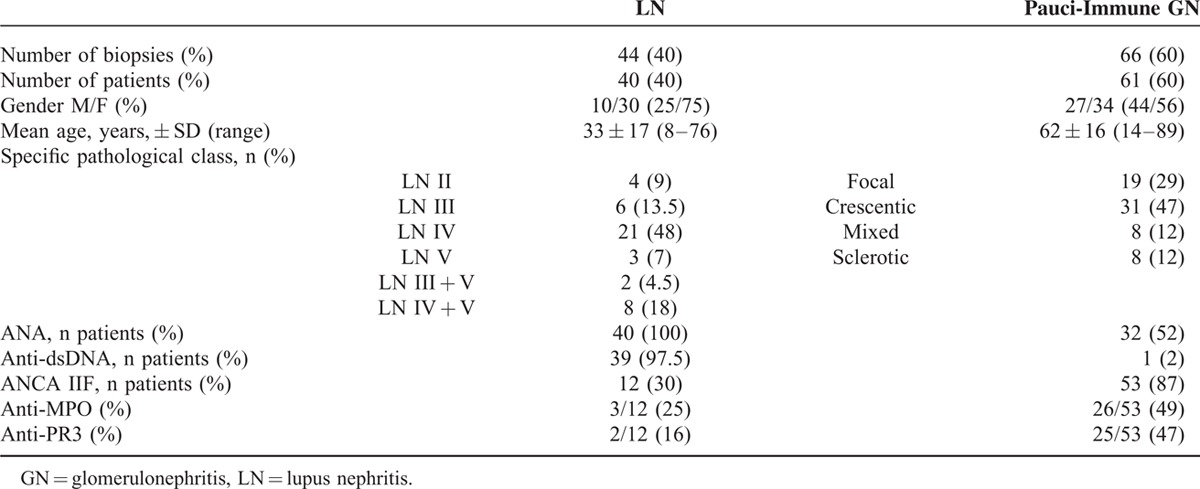
Characteristics of Patients With Lupus Nephritis (LN) and Pauci-Immune Glomerulonephritis (GN) From an Independent Cohort (110 Renal Biopsies Performed in 101 Patients)

Serum ANCA antibodies were detected by IIF at the time of a kidney biopsy in 12 (30%) patients with LN. Fluorescence was perinuclear (p-ANCA) in 9 patients and cytoplasmic (c-ANCA) in 3. Anti-MPO and anti-PR3 antibodies were detected by ELISA in, respectively, 3 and 2 patients.

Kidney biopsies of LN were found in 2 groups according to ANCA IIF positivity. The pathological characteristics are summarized in Table 5. There was no significant clinical or pathological difference in SLE patients according to the presence or absence of ANCA. No patient with LN and positive ANCA fulfilled the AAV classification criteria.

**TABLE 5:**
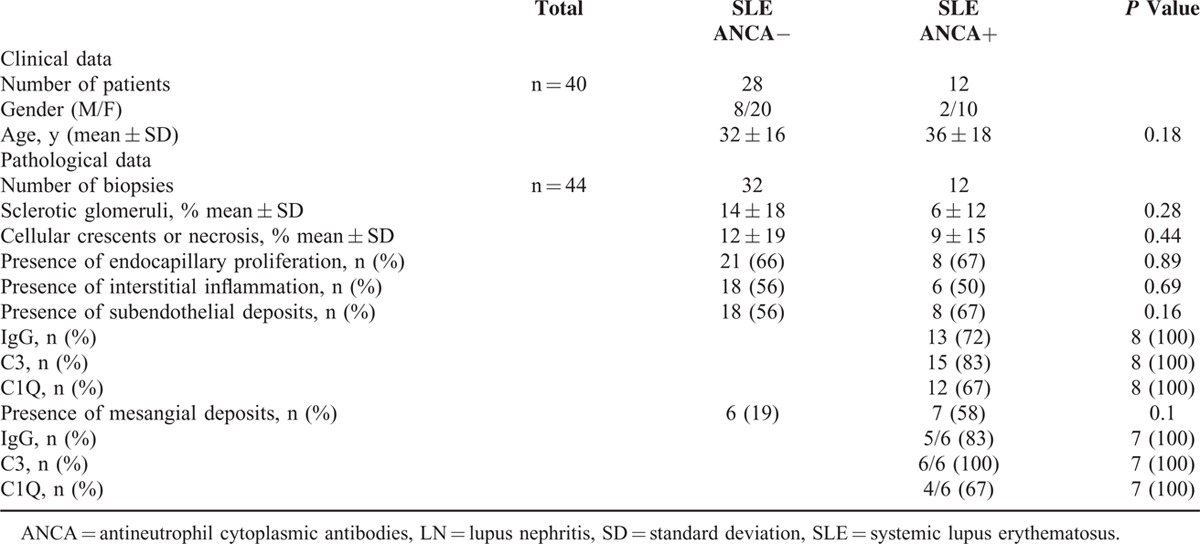
Clinical and Pathological Characteristics of Patients With Lupus Nephritis (LN) According to ANCA Status

Serum ANA was detected at the time of a renal biopsy in 36 (52%) cases of pauci-immune GN, with a mean titer of 1/700. Fluorescence was speckled in 89% and homogenous in 11% of cases. Antiextractable nuclear-antigen antibodies were positive in 3 cases (9%), including anti-SSA/SSB in 2 cases and anti-Sm in 1 case. Anti-dsDNA antibodies were positive in 1 patient and were at the threshold positivity value in 7 patients (11%). Low complement fractions were reported in 2 cases (3%). Kidney biopsies of pauci-immune GN were displayed in 2 groups according to serum ANA positivity. The pathological characteristics are summarized in Table 6. There was no significant clinical or pathological difference in patients with pauci-immune GN according to the presence or absence of ANA.

**TABLE 6:**
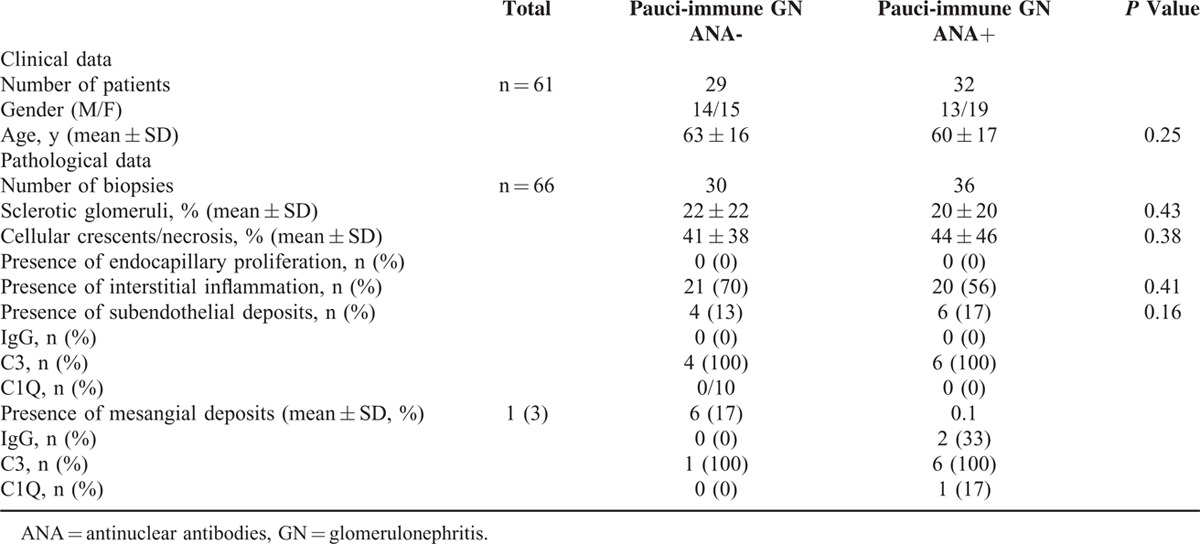
Clinical and Pathological Characteristics of Patients With Pauci-Immune Glomerulonephritis (GN) According to ANA Status

**FIGURE 1:**
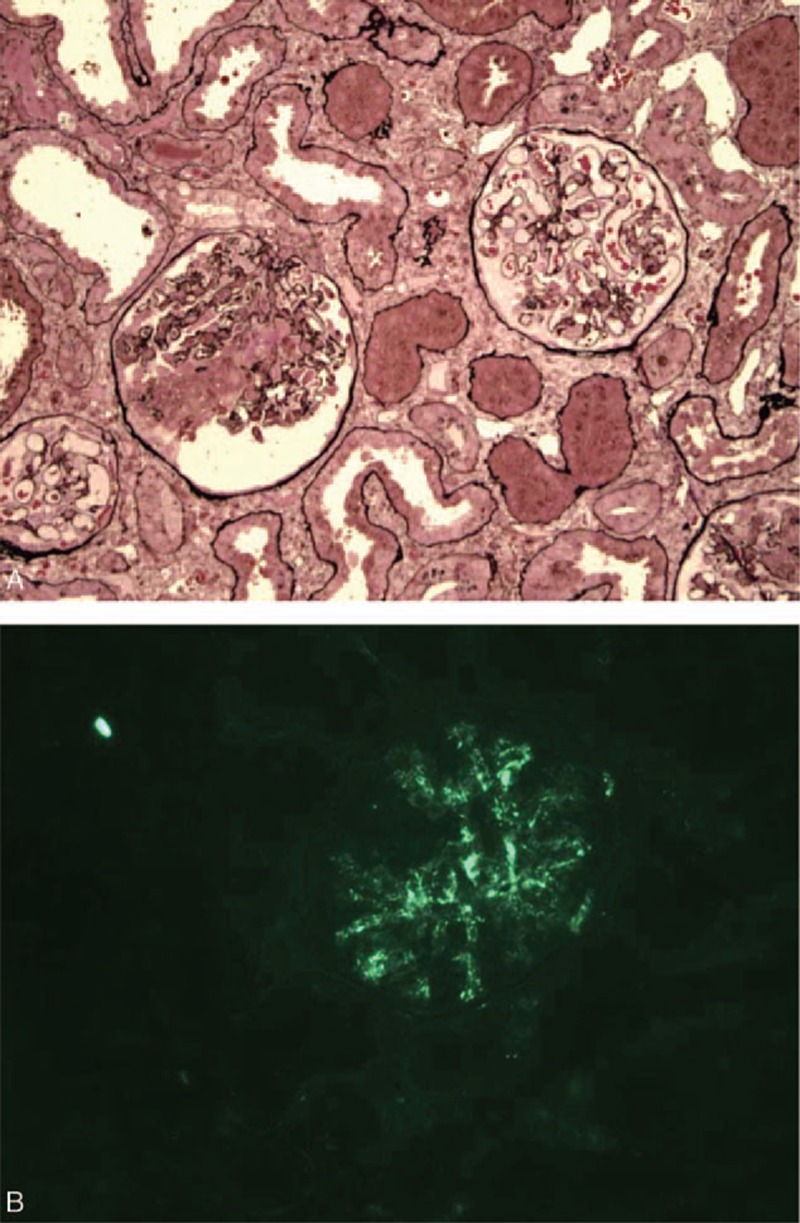
Kidney biopsy from a patient with SLE/AAV overlap syndrome. Patient 7 presented with rapidly progressive glomerulonephritis (GN) following anti-TNF therapy for polyarthritis, with positive ANCA and anti-MPO antibodies. Renal pathology shows crescentic glomerulonephritis compatible with pauci-immune GN, but with unexpected mesangial enlargement and immune-complex deposits in immunofluorescence analysis, which led to a search for the SLE criteria and a diagnosis of SLE/AAV overlap syndrome. (A) Optical analysis: the glomerulus on the left shows fibrinoid necrosis and a cellular crescent, whereas the glomerulus on the right shows discrete mesangial enlargement, without proliferation (Jones methenamine silver, ×100 magnification). (B) Immunofluorescence analysis: the presence of diffuse mesangial immune-complex deposits of IgG (+), IgM (±), C3 (+), and C1q (±) (IgG + shown here, ×200 magnification). AAV = ANCA-associated vasculitis, GN =  glomerulonephritis, MPO = myeloperoxidase, SLE = systemic lupus erythematosus.

Among patients diagnosed with pauci-immune GN, only 2 female patients fulfilled the ACR classification criteria for SLE and were included in the national survey cohort for SLE/AAV overlap syndrome (patients 1 and 7). The first patient (1) had scarred pauci-immune GN without active lesions. The second patient (7) presented with unexpected mesangial immune-complex deposits (IgG, IgM, C3, C1q) on a kidney biopsy, with 26% necrotic/crescentic glomeruli and no endocapillary or mesangial proliferation (Figure 1). Both had an inflammatory interstitial infiltrate. Their clinical and biological characteristics are described in Table 1  .

Overall, among the 101 patients from this cohort, 57 (56.5%) had typical LN or typical pauci-immune GN, without overlapping antibodies; 12 (12%) had typical LN and positive ANCA antibodies; 30 (29.5%) had typical pauci-immune GN and positive ANA antibodies; and only 2 (2%) patients fulfilled the classification criteria for both SLE and AAV, corresponding to the SLE/AAV overlap syndrome.

## DISCUSSION

Combining the results from a national survey across referral centers and a systematic literature review, we report here on what is, to the best of our knowledge, the largest series of patients with histologically proven GN and SLE/AAV overlap syndrome.

SLE and AAV are rare autoimmune diseases that share clinical symptoms, such as arthritis and cutaneous lesions, as well as possibly severe renal involvement. Whereas SLE and AAV can be easily distinguished by demographic characteristics, an autoantibody profile and a renal pathology in most cases, some patients display mixed patterns responding to both SLE and AAV classification criteria. Altogether, patients with SLE/AAV overlap syndrome are mostly female, with a wide range of age distributions at the onset of SLE or AAV. The presentation of AAV is MPA-like in most patients, with articular, cutaneous and renal involvement, as well as IAH in some patients, concordant with the exclusive positivity of anti-MPO antibodies.^24^ Although ear–nose–throat involvement was present in some patients, no granuloma has been documented on renal pathology, and no patient with SLE/AAV overlap from our study or the literature had anti-PR3 antibodies. Renal pathology, classified as either LN or pauci-immune GN according to the predominant type of lesion, displayed overlapping lesions in some cases.

Crescentic GN is not rare in SLE: it accounts for 10% of biopsy-proven LN and 20% of class IV-G LN.^24^ The presence of ANCA antibodies has been reported in up to 20% of SLE patients in the literature,^25^ but their prevalence varies between IIF and ELISA.^26^ Interestingly, anti-MPO ANCA antibodies are more frequently encountered in patients with crescentic LN than in patients with noncrescentic LN.^23^ Some authors also report a correlation between p-ANCA and crescent formation^27^ and a possible association between other p-ANCA, namely anticathepsin G antibodies and the development of LN, suggesting a potential pathogenic role for p-ANCA in crescent formation.^7,8,28^

The ISN/RPS 2003 classification of LN individualizes segmental from a global pattern of class IV LN, suggesting that they may have a different pathogenesis and prognosis.^5^ An association of segmental forms of proliferative LN with crescents and necrosis has been reported by several authors, whereas global patterns are associated with abundant immune-complex deposits.^29–31^ Although some segmental forms of proliferative LN are considered similar to pauci-immune GN, ANCA status is not available for most patients in these cohorts.^29^

Likewise, although ANCA-associated GN is defined as pauci-immune, the presence of immune-complex deposits has been documented by electron microscopy in 18 and 54% of patients from 2 large cohorts, without mention of ANA status.^32,33^

We assessed the prevalence of overlapping antibodies and their association with renal pathological features in a cohort of biopsy-proven GN. The prevalence of ANCA in the LN cohort is consistent with data from the literature, with less common MPO or PR3 specificity. We describe, for the first time, a high prevalence of ANA in patients with pauci-immune GN. However, the presence of overlapping antibodies in LN or pauci-immune GN was not associated with the proportion of crescentic glomeruli, or the presence of endocapillary proliferation, interstitial inflammation, or immune deposits in this cohort. Finally, we do not find a pathological relevance for overlapping antibodies, which highlights the difference between true SLE/AAV overlap syndrome and the more common finding of ANCA positivity in LN.

This study has some limitations. The absence of electron microscopy of kidney biopsies prevented detailed analysis of localization of immune-complex deposits. Also, SLE/AAV overlap syndrome may have been underestimated in this study due to the small number of cases. Indeed, because of the stringent inclusion criteria, with exclusion of patients with exclusively extra-renal manifestations of SLE and AAV, only 8 patients with biopsy-proven GN were identified after screening a large database that included 3300 cases of vasculitis, of which 1990 were AAV from the FSGV registry.

Although this study was not designed to address the pathophysiology of SLE/AAV overlap syndrome, some hypotheses can be made. First, the co-occurrence of SLE and AAV may be part of a global poly-autoimmunity in some patients, as suggested by the frequent association with a third autoimmune disease in our cohort. The shift and diversification of autoantibody targets during the course of autoimmune diseases has been defined as epitope spreading.^34^ This phenomenon has been studied in SLE, in particular, and may explain the numerous autoantibodies encountered in SLE and the frequent association of SLE with antiphospholipid syndrome or Sjögren's syndrome.^1,35^ It has been also studied in AAV, where the spread of the ANCA epitope may differentiate patients with full-blown vasculitis from patients with positive antibodies but with no clinical manifestations.^36^ The association of AAV with another autoimmune disease, although rare, has been reported.^37,38^

The second hypothesis is that drug exposure may have participated in some patients. The presence of anti-MPO antibodies has already been reported in patients with drug-induced SLE.^39^ In the literature, 4 cases of SLE/AAV overlap syndrome had been exposed to hydralazine or thioridazine, which have been associated with drug-induced SLE or AAV.^39–41^ One additional patient from the present report had received etanercept, a drug associated with SLE autoantibody generation and sometimes clinical symptoms of SLE.^40^ The shared genetic variants, identified through genome-wide association studies, may predispose patients to the development of several immune-mediated disease.^42^ Finally, although neutrophil activation is the hallmark of AAV,^43^ gene-expression studies have shown a strong neutrophil signature in SLE.^44–46^ The release of neutrophil extracellular traps,^47^ in particular, may participate in the pathogenesis of both AAV-related GN and lupus nephritis.^48,49^

## CONCLUSION

The association of SLE and AAV with biopsy-proven GN seems rare, but not fortuitous. Patients with SLE/AAV overlap syndrome are mostly female, present with a severe clinical presentation (rapidly progressive GN and frequent pulmonary involvement), and have both ANA and anti-MPO antibodies. Future studies are needed to investigate the possible shared immune pathogenesis that may facilitate the co-occurrence of SLE and AAV in these patients and so lead to new therapeutic options.

## Supplementary Material

Supplemental Digital Content
