# Supplementary material for: Systemic Lupus Erythematosus and Antineutrophil Cytoplasmic Antibody-Associated Vasculitis Overlap Syndrome in Patients With Biopsy-Proven Glomerulonephritis
Source: Medicine (Baltimore). 2016 Jun 3;95(22):e3748. doi: 10.1097/MD.0000000000003748 (PMC4900711; doi:10.1097/MD.0000000000003748)
Supplement: Supplemental Digital Content [file medi-95-e3748-s001.doc]

**SUPPLEMENTAL METHODS**

1. **Remission criteria in patients with SLE/AAV overlap syndrome**

When the latest diagnosis was AAV^1^, complete remission was defined by BVAS = 0; response was defined by a 50% reduction in BVAS and the absence of new AAV manifestation; relapse was defined by the re-occurrence or new onset of active disease; refractory disease was defined by unchanged or increased BVAS after 4 weeks of standard therapy.

When the latest diagnosis was SLE^2,3^, complete remission was defined by a urinary protein excretion < 0.3 g/day, with normal urinary sediment, serum albumin, and renal function. Partial remission was defined by the presence of any of the following features: decrease of serum creatinine ≤130 µmol/L in patients with a baseline serum creatinine between 130-260 µmol/L; decrease of serum creatinine by 50% for patients with a baseline serum creatinine >260 µmol/L; decrease of urinary protein excretion by 50% and <3 g/24h, with a serum albumin >3 g/dL and stable renal function. Treatment failure was defined as the absence of remission. Relapse was defined by the re-occurrence of proteinuria (or significant increase in baseline proteinuria) and/or active urinary sediment in a patient previously in remission, and was documented by renal biopsy in most cases.

1. **Pathological analysis**

Kidney biopsy specimens were examined under light microscopy, and with direct Immunofluorescence (IF) with a full panel (IgG, IgM, IgA, C3, C1q, κ and λ light chains). Pathologists were blinded to the results of serum auto-antibodies. The IF for mesangial or subendothelial deposition of immunoglobulin IgG, IgA, IgM, C3, C1q was semi-quantitatively graded from 0 to 4+ according to the intensity of fluorescence. Deposits of immunoglobulins were considered positive when intensity ≥ 1+. Interstitial inflammatory infiltrate was semi-quantitatively evaluated with the Banff transplant scoring, i.e., ≤ 5% (absent), 6 – 25% (mild), 26 – 50% (moderate), and > 50% (severe). Glomerular tuft necrosis was defined by disruption of the glomerular basal membrane or fragmentation of nuclei, with or without fibrin. The percentages of glomeruli with cellular or fibro-cellular crescents and with endocapillary proliferation were calculated from the total number of glomeruli. The percentage of sclerotic glomeruli was calculated.

LN was defined by mesangial hypercellularity, and/or endo/extra capillary GN, and/or membranous glomerulonephritis with mesangial or subendothelial immune complex deposits, associated with at least 3 ACR criteria of SLE. LN lesions were classified according to the 2003 revised classification of ISN/RPS^4^. Pauci-immune GN was defined by the presence of glomerular necrosis or crescents in ≥ 1 glomerulus, without significant immune complex deposits, associated or not with the presence of ANCA antibodies.

In patients with LN, the presence of crescents and/or necrosis, endocapillary proliferation and the intensity of inflammatory interstitial infiltration were compared between kidney biopsies of patients with positive versus negative ANCA antibodies.

In patients with pauci-immune GN, the presence of mesangial or subendothelial deposits and the intensity of inflammatory interstitial infiltration were compared between Kidney biopsies of patients with positive versus negative ANA.

1. **Immunological analysis**

The presence of ANA and their staining pattern was assessed by Indirect IF (IIF) and considered positive when ≥ 1/160. Anti-ENA were detected and quantified by enzyme link immunosorbent assay (ELISA). The presence and intensity of anti-dsDNA antibodies was determined by Farr test and ELISA. Anti-dsDNA positivity threshold was 15 IU/mL. ANCA antibodies and their staining pattern (p or c-ANCA (perinuclear, cytoplasmic) were detected by IIF on ethanol-fixed human neutrophil slides (Euroimmun, Germany), and their titer and specificity (anti-MPO or PR3) were determined by ELISA (Bioadvance, France). Complement fractions C3 and C4 were measured by immunonephelometry on BN system (Siemens Diagnostics, Germany). Total complement activity (CH50) was evaluated by determining the lysis of erythrocytes on Behring Coagulation Timer (BCT, Siemens Diagnostics). The presence of cryoglobulin was determined by cryocrit and further characterized by immuno-blotting.

**References**

1. Hellmich B, Flossmann O, Gross WL, et al. EULAR recommendations for conducting clinical studies and/or clinical trials in systemic vasculitis: focus on anti-neutrophil cytoplasm antibody-associated vasculitis. Ann Rheum Dis. 2007;66:605-17.

2. Wang J, Hu W, Xie H, et al. Induction therapies for class IV lupus nephritis with non-inflammatory necrotizing vasculopathy: mycophenolate mofetil or intravenous cyclophosphamide. Lupus. 2007;16:707-12.

3. Contreras G, Pardo V, Leclercq B, et al. Sequential therapies for proliferative lupus nephritis. N Engl J Med. 2004;350:971-80.

4. Weening JJ, D'Agati VD, Schwartz MM, et al. The classification of glomerulonephritis in systemic lupus erythematosus revisited. Kidney Int. 2004;65:521-30.
